# The Clinical Society

**Published:** 1896-11-21

**Authors:** 


### Medical Progress and Hospital Clinics.

[*The Editor will be glad to receive offers of co-operation and contributions from members of the profession. All letters should be addressed to* The Editor, at the Office, 28 & 29, Southampton Street, Strand, London, W.C.]

### THE CLINICAL SOCIETY.

The treatment of suppurative pericarditis is a matter of great interest. The tendency of the surgical instinct is to deal with it as with any other collection of pus—to open and to drain it. On the other hand, men have hesitated to do anything that might interfere with the action of an organ so important to the maintenance of life; and although tapping of the pericardium has been performed in a large number of cases, the treatment on ordinary surgical principles of purulent effusion in its cavity has been comparatively rarely undertaken. Yet the neighbouring cavity—the pleura—has been attacked with the utmost freedom, and with the most satisfactory results, notwithstanding that, so far as the physics of the matter are concerned, the heart ought to be able to act with an open pericardium far better than the lung can do with an open pleura. That, in fact, this is so, and that whatever other difficulties there may be in the way the free opening of the pericardium does not interfere with the proper action of the heart, is shown by a case related to the Clinical Society by Mr. H. B. Robinson on November 13th.

The patient, a lad aged sixteen, was attacked with sore throat, which was soon followed by pain in the left side and swelling in the left wrist. The day after he was first seen a pericardial rub was perceived. Ten days later the heart's action was found to be interfered with, and the area of the cardiac dulness was enlarged both upwards and outwards. A diagnosis of fluid in the pericardium, probably pus, was arrived at, although it was thought possible that it might be a localised pleurisy.

On making an exploratory puncture no fluid was at first discovered, but on inserting a longer needle pus was withdrawn, which was thought to come from the position of the pericardium. An incision three inches in length was then made along the upper border of the sixth rib, and a piece of this rib two inches long was removed. The pleura was found to be dry, and the lung was adherent to the pericardium, which bulged towards the incision. On cutting into the pericardium a flood of pus escaped, and the embarrassment of the heart was at once relieved. Two quarts of pus were removed, much of it being very flakey. A long drainage tube was then inserted and stitched to the edge of the wound. Except for a slight rise of temperature in connection with some suppuration which occurred over the left wrist, recovery was uninterrupted. No pus formed in the pleura, owing probably to the adhesions of the lung shutting off the general pleural sac. The drainage tube was gradually shortened, and was finally removed sixty-one days after the operation, but it was uncertain how long it had remained actually in the pericardium.

Since then the patient had undergone complete recovery. He could ride the bicycle and walk ten miles. There was no interference with the normal respiratory sounds, no friction sounds were to be heard, and, what was curious, there was no restriction of the interspaces during the cardiac systole.

Two questions of some interest arise in regard to this case; first as to the best point for the incision, next as to the desirability of irrigating the pericardium. More than one speaker bore testimony to the curdy nature of the pus contained in such cases, and to the apparent desirability of using irrigation with the object of removing it; but it was pointed out by Mr. Parker that if the process were so conducted as to produce any pressure on the heart, the mere irrigation of the pericardium might be the last straw, and might lead to immediate death. In regard to this point, Mr. Parker related a case in which death had occurred during irrigation after opening the pericardium. It is to be noted that in this instance, the opening was made much nearer the central line than in Mr. Robinson's case. It would seem, in fact, that unless very great care is taken to ensure the rapid escape of the fluid, any attempt to irrigate the pericardium is apt to produce one of the evils which the incision was intended to remove, viz., pressure on the surface of the heart and obstruction to its diastole. Nevertheless, experience in the dead house shows how largely fluid may accumulate in the deeper recesses of the pericardium, and how little it may tend to drain away, and it would appear that this question of irrigation is intimately connected with the other question of the position of the opening, for while a free lateral opening would probably lessen the one great danger of irrigation, viz., pressure; it would also, to a large extent, lessen the necessity for its performance, in consequence of the far better drain which would be provided. On the one hand, we have Mr. Parker's suggestion that drainage in front is the most direct; on the other, that of Mr. Robinson that drainage at the side is the most efficient, and that if the pleura should become infected, what drains the one will drain the other.

In regard to the question of operating at all, Dr. Sansom's observation must be remembered, to the effect, viz., that in such cases the pericarditis is usually but part of a wide infection, and that recovery cannot always be expected to result from even the most successful measures to remove the pus.

